# Risk Factors and Outcome of Pulmonary Artery Stenting After Bidirectional Cavopulmonary Connection (BDCPC) in Single Ventricle Circulation

**DOI:** 10.1007/s00246-023-03229-3

**Published:** 2023-07-15

**Authors:** Alessia Callegari, Jana Logoteta, Walter Knirsch, Robert Cesnjevar, Hitendu Dave, Oliver Kretschmar, Daniel Quandt

**Affiliations:** 1 Pediatric Heart Centre, Division of Pediatric Cardiology, University Children’s Hospital Zurich, Steinwiesstrasse 75, 8032 Zurich, Switzerland; 2 Department of Congenital Cardiothoracic Surgery, University Children’s Hospital Zurich, Zurich, Switzerland; 3 Children’s Research Centre, Zurich, Switzerland; 4 University of Zurich, Zurich, Switzerland

**Keywords:** Single ventricle, PA-stenting, Bidirectional cavopulmonary connection, Pulmonary arteries, Pulmonary arteries growth

## Abstract

After bidirectional cavopulmonary connection (BDCPC) central pulmonary arteries (PAs) of single ventricle (SV) patients can be affected by stenosis or even closure. Aim of this study is to compare SV patients with and without PA-stent implantation post-BDCPC regarding risk factors for stent implantation and outcome. Single center, retrospective (2006–2021) study of 136 SV consecutive patients with and without PA-stent implantation post-BDCPC. Patient characteristics, risk factors for PA-stent implantation and PA growth were assessed comparing angiographic data pre-BDCPC and pre-TCPC. A total of 40/136 (29%) patients underwent PA-stent implantation at median (IQR) 14 (1.1–39.0) days post-BDCPC. 37/40 (92.5%) underwent LPA-stenting. Multiple regression analysis showed single LV patients to receive less likely PA-stents than single RV patients (OR 0.41; *p* = 0.05). Reduced LPA/BSA (mm/m2) and larger diameter of neo-ascending aorta pre-BDCPC were associated with an increased likelihood of PA-stent implantation post-BDCPC (OR 0.89, *p* = 0.03; OR 1.05, *p* = 0.001). Stent re-dilatation was performed in 36/40 (89%) after 1 (0.8–1.5) year. Pulmonary artery diameters pre-BDCPC were lower in the PA-stent group: McGoon (*p* < 0.001), Nakata (*p* < 0.001). Indexed pulmonary artery diameters increased equally in both groups but remained lower pre-TCPC in the PA-stent group: McGoon (*p* < 0.001), Nakata (*p* = 0.009), and Lower Lobe Index (*p* = 0.003). LPA and RPA grew symmetrically in both groups. Single RV, larger neo-ascending aorta, and small LPA pre- BDCPC are independent risk factors for PA-stent implantation post-BDCPC. Pulmonary artery diameters after PA-stent implantation and stent re-dilatation showed significant growth together with the contralateral side, but the PA-system remained symmetrically smaller in the stent group.

## Introduction

Pulmonary artery growth, a sufficient cross-sectional area, and low resistance of the pulmonary vascular system strongly influence long-term outcome of patients with single ventricle (SV) physiologies undergoing Fontan completion [1–4].

Vessel stenosis, vessel distortion, vessel obstruction, or hypoplasia of the central and peripheral pulmonary arteries (PAs), especially of the left pulmonary artery (LPA), affect almost half of the SV patients after bidirectional cavopulmonary connection (BDCPC) [5–10]. This may be attributed to an *obstruction*, which is due to external compression by the reconstructed aorta (anteriorly) or left main bronchus (posteriorly) [7, 8, 11, 12], or a *stenosis*, which results from abnormal surgical connections, ductal constriction, or as a consequence of surgical patch reconstruction during BDCPC [4].

Since a certain degree of compression is almost always present [7, 8, 11, 12], surgical management of these lesions may be limited by the proximity to the surrounding structures [7]. On the other hand, a PA-stent fixes the branch PA to a static non-pulsatile and non-compressible state [8, 12]. Percutaneous PA-stent implantation has become the preferred treatment option for these patients, even in the early postoperative period [5, 12–17]. Nevertheless, the stent implanted during infancy does not grow and needs to be re-dilated or even removed surgically during follow up [6–8].

Improvement of the size and flow to the PAs, even in mild cases of stenosis or mild compression, can improve the hemodynamics and potentially maximize the potential for future symmetrical growth of the PAs by equalizing blood flow to both lungs [7, 18]. Therefore prompt relief of pulmonary stenosis is essential [5, 6]. Identifying risk factors, ideally making a prediction of patients at risk for later PA-stent implantation, as well as timely recognition, and interventional therapy of these lesions, potentially also as a hybrid procedure during BDCPC, may significantly improve outcome.

Little has been published on PA-stent implantation after BDCPC in SV patients [5, 6, 8, 11, 12], while outcome in relation to pulmonary artery growth has barely been assessed [19].

The aim of this study is to identity patients at risk for PA-stent implantation after BDCPC by evaluating the periinterventional course and assessing the dimensions and growth of the PAs comparing angiographic data pre-BDCPC and pre-total cavopulmonary connection (TCPC) in a group of patients that required PA-stent implantation versus a group that did not require PA-stent implantation.

## Methods

### Study Design and Patients’ Selection

This is a single center, retrospective, longitudinal analysis of all consecutive SV patients undergoing an invasive hemodynamic and angiographic evaluation pre-BDCPC and pre-TCPC between 2006 and 2021. At least two cardiac catheterizations were performed in each patient (one pre-BDCPC and one pre-TCPC) in accordance with the institutional standards of the University Children’s Hospital Zurich at that time. We excluded all patients with only one cardiac catheterization (prior to BDCPC or prior to TCPC), congenital abnormalities of the pulmonary arteries (specifically, patients with pulmonal atresia and major aortopulmonary collateral arteries after surgical unifocalization), or a pulsatile BDCPC physiology (with a Shunt and a BDCPC) or a delayed BDCPC with a second shunt placement, or patients with a temporary PA-stent (implanted after BDCPC but surgically explanted prior to TCPC).

**Fig. 1:**
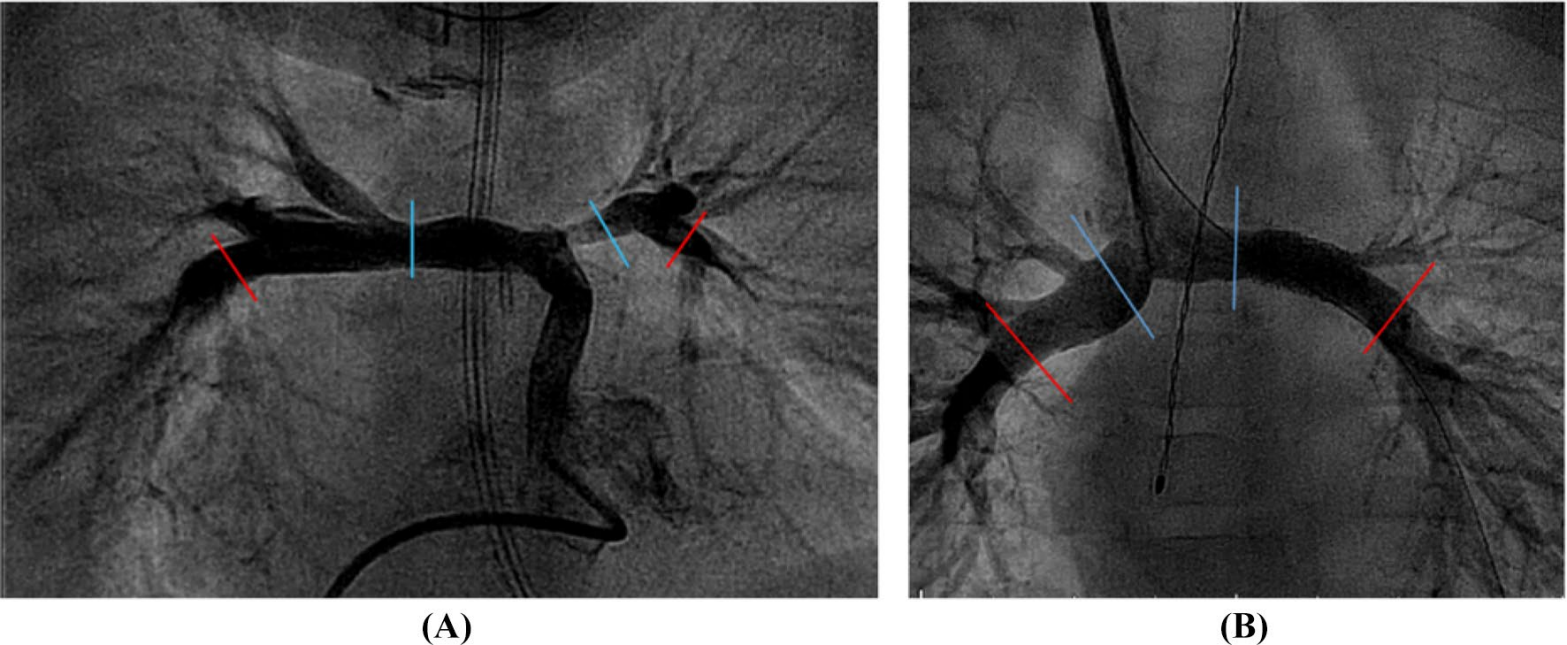
Angiographic measurement sites for the central pulmonary arteries (blue) and lower lobes arteries (red) prior to BDCPC (**A**) and prior to TCPC (**B**)

Demographic data, underlying congenital heart disease, preceding congenital cardiac surgeries or cardiac catheter interventions, indications for cardiac catheterization leading to PA-stent implantation, peri-/post-procedural data, and clinical course until TCPC were analyzed. To evaluate and compare the growth of the pulmonary arteries the Nakata, McGoon, and Total Lower Lobe indices [20, 21]; the LPA and right pulmonary artery (RPA) dimensions normalized for body surface area (BSA); the ratio RPA to LPA and right lower lobe (RLL) to left lower lobe (LLL), and the individual relative growth (%) of each of these parameters were measured angiographically [20, 21]. The measurement sites are shown in Fig. 1. The angiographic dimensions of the (neo-) ascending aorta (at LPA level on a lateral projection) were also assessed and normalized for BSA. Data was analyzed forming two groups: patients undergoing PA-stent implantation after BDCPC (group 1) and patients not undergoing a PA-stent implantation after BDCPC (group 2).

### Cardiac Catheterization Procedures

Cardiac catheterizations were performed under general anesthesia. Patients received a 100 IU/kg bolus of heparin intravenously, if they were not anticoagulated prior to cardiac catheterization; otherwise, peri-procedural anticoagulation management was monitored, and titrated measuring activated clotting time. Standard intravenous antibiotic prophylaxis (cephalosporin 2nd generation) was given if a stent was implanted. Biplane angiography was used in all procedures. Anticoagulation continued after catheterization using subcutaneous low-molecular-weight heparin for 24–36 h and was then switched to oral acetylsalicylic acid (3-5 mg/kg/day) monotherapy.

### Statistics

Statistical analysis was performed using SPSS 27.0.0 (SPSS Inc, IBM Company, Chicago Illinois/USA). Continuous variables are expressed as median (IQR), categorical data as counts and percentages. Group comparison was performed using two-tailed sample t-tests and Levene’s test for equality of variance was used to analyze, if the variability in the two groups was significantly different. Ordinal, nominal, and dichotomic variables were evaluated with contingency tables and compared with chi-square-tests or Kolmogorov–Smirnov analyses. Predictability of the continuous variables was evaluated by means of Pearson correlations and stepwise multiple regressions, with the criteria probability of F to enter *p* < 0.05, probability of F to be removed *p* > 0.1. Binomial logistic regression and Cox stepwise linear regressions were used for dichotomic variables. Significance is defined by values of *p* < 0.05. A prediction model to estimate the predicted probability of LPA-stent implantation after BDCPC was calculated with the coefficients of the logistic regression model that included the independent predictors of the necessity of LPA-stent implantation after BDCPC. This logistic regression model generated the coefficient of a formula to predict a logit transformation of the probability of LPA-stent implantation after BDCPC. The results of the logit transformation were then converted in predicted probability using the exponential function of the Euler's number. The specificity and sensibility of this model was tested on a ROC-curve.

### Ethics

All data were primarily obtained for medical purposes, with informed consent for the performed cardiac catheterization procedure. The study design fulfills the guidelines of the declaration of Helsinki regarding ethical principles for medical research involving human subjects. The study was approved by the institutional ethical board (KEK: 2017–00564).

## Results

### Patients

A total of 136 patients, 56 (41%) females, were included in the study. A single right ventricle (RV) was present in 69 (51%) of the patients. The complete anatomical characteristics of the patients and surgical/interventional approach at stage I are shown in Table 1.

**Table 1:** Characteristics of the complete patients’ cohort as well as comparison of group 1 (with PA-stent) and group 2 (without PA-stent) referring to baseline anatomy and procedures at stage I and II

Patients		All patients		With PA-stent		Without PA-stent		
		n	%	n	%	n	%	p
Total		136	100	40	29	96	71	/
Sex	female	56	41	14	25	42	75	0.89
	male	80	59	26	33	54	67	/
Ventricle	Left ventricle	65	48	13	20	52	80	**0.03**
	Right ventricle	69	51	27	39	42	61	/
	Right&left ventricle	2	1	0	0	2	100	/
Aortic arch	left	131	96	38	30	93	70	/
	right	5	4	2	40	3	60	/
Diagnosis	dysbalanced AVSD	10	7	1	1	9	90	/
	DILV	16	12	3	19	13	81	/
	DILV, aortic arch anomaly	5	4	2	40	3	60	/
	DORV	17	13	3	18	14	82	/
	DORV, aortic arch anomaly	2	2	0	0	2	100	/
	Ebstein anomaly	1	1	1	100	0	0	/
	HLHS and HLHS-complex	44	32	25	57	19	43	** < 0.001**
	PA (VSD or IVS)	18	13	3	16	15	84	/
	TA	19	14	3	16	16	84	/
	TA, aortic arch anomaly	1	1	1	100	0	0	/
	TGA, VSD	3	2	0	0	3	98	/

Stage I		n	%	n	%	n	%	p
Norwood I		39	29	16	41	23	59	0.06
	with mBTS	30	77	12	40	18	60	0.31
	with AP-Shunt	2	5	1	50	1	50	/
	with Sano-Shunt	7	18	3	43	4	57	/
None		11	8	0	0	11	100	**0.05**
Giessen-approach		26	19	12	46	14	54	0.07
Central PA-Banding		26	19	3	12	23	88	**0.04**
Shunt	Total	34	25	8	23	26	77	0.51
	mBTS	18	52	2	12	16	88	0.1
	PDA-Stent	5	14	1	20	4	80	/
	Aorto-pulmonary-shunt	10	32	5	50	5	50	/
	Sano shunt	1	2	1	100	0	0	/

Stage II		median/n	range/%	median/n	range/%	median/n	range/%	p
Age	weeks	20.1	15; 26.3	17	13; 24.5	22.4	27.7; 26.4	**0.009**
Procedure	BDCPC	92	68	23	25	69	75	**0.003**
	Bilateral BDCPC	8	6	2	25	6	75	0.9
	Comprehensive stage I-II	25	18	11	44	14	56	0.06
	BDCPC + DKS anastomosis	11	8	2	18	9	82	0.6
	Additional PA-Patch	36	27	10	28	26	72	0.8

Statistically significant results are given in bold

*AVSD* atrioventricular septal defect, *DILV* double inlet left ventricle, *DORV* double outlet right ventricle, *HLHS* hypoplastic left heart syndrome, *PA*: pulmonary atresia, *VSD* ventricular septal defect, *IVS* intact ventricular septum, *TA* tricuspid atresia, *TGA* transposition of the great arteries, *mBTS* modified Blalock-Taussig shunt, *PA* pulmonary arteries, *PDA* patent ductus arteriosus, *BDCPC* bidirectional cavopulmonary connection, *DKS* damus-kaye-stansel

### Peri-and Postinterventional Course

**Fig. 2:**
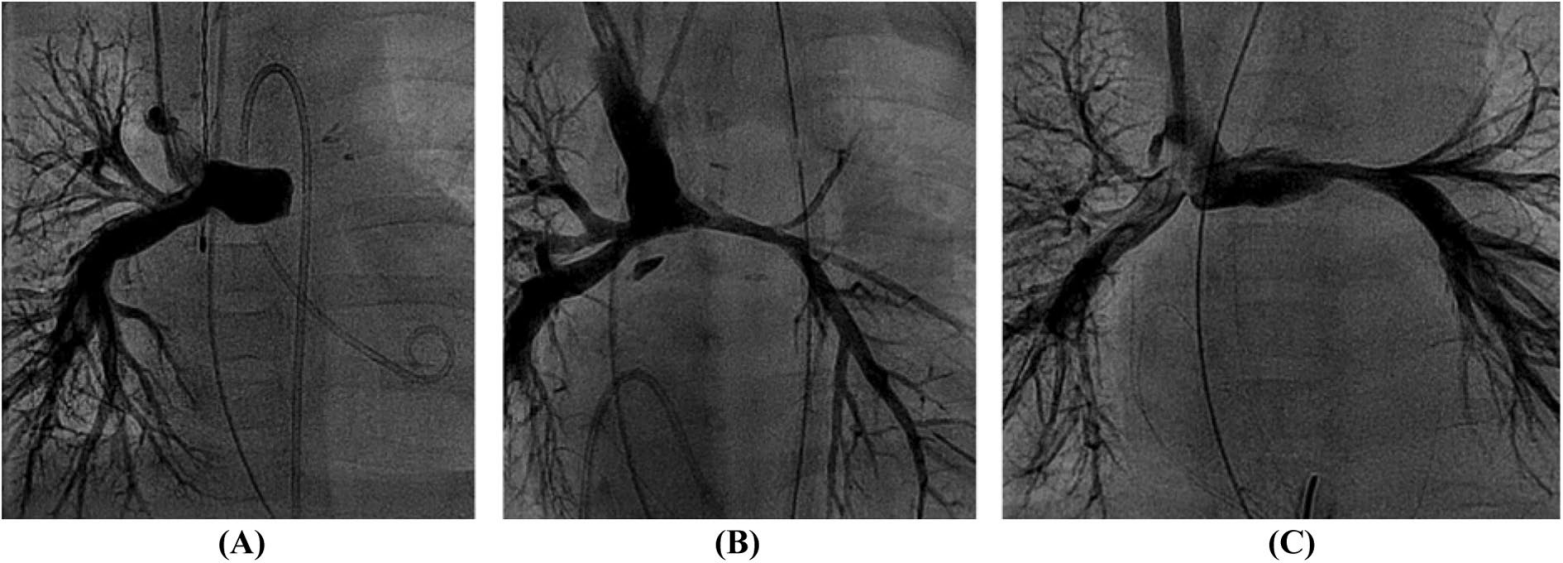
Main intraprocedural findings at PA-stent implantation after BDCPC were **A** suspected LPA compression from the ascending aorta (16/40, 40%); **B** LPA hypoplasia (12/40, 30%); **C** stenosis and vascular torsion after surgical anastomosis (11/40, 27.5%); RPA torsion (1/40, 2.5%)

A total of 40/136 (29%) patients underwent PA-stent implantation at median (IQR) 14 (1.1–39) days post-BDCPC at an age of 5 (3–5) months. 37/40 (92.5%) underwent LPA-stenting, 3/40 (7.5%) (2 of which with right aortic arch) RPA-stenting. Reasons for cardiac catheterization were echocardiographically suspected LPA stenosis in 26/40 (65%), RPA stenosis in 1/40 (3%), or low oxygen saturation in 13/40 (33%). Main intraprocedural findings (Fig. 2) at PA-stent implantation after BDCPC were classified as external LPA compression from the ascending aorta (16/40, 40%; in two cases with a complete occlusion of the LPA lumen); LPA hypoplasia (12/40, 30%); LPA stenosis and vascular torsion after surgical anastomosis (11/40, 27.5%); and RPA torsion (1/40, 2.5%). In 7/40 cases (18%) more than one stent was used. No coronary stents were used, and all stents were pre-mounted, balloon expandable, bare metal stents with a closed cell design (e.g., Cordis® Palmaz blue or Palmaz Genesis, or Cook® Formula, or Bentley BeGrow™ stents [22]). Median (IQR) stent diameter was 6 (6–7) mm and length 16 (15–18) mm. Increase of absolute vessel diameter after PA-stent implantation was median (IQR) 3.3 (3.1–4.4) mm and stent-to-stenosis ratio was median 2.4 (IQR 1.9–3.7). Three periinterventional complications occurred: a tracheal bleeding in one patient, and two branch PA dissections requiring surgical intervention. The cause of the dissection was a balloon-dilatation (preceding the stent implantation) across circumferential suture lines in the early postoperative period in the first patient and a dissection after sheath insertion in the second patient. All patients recovered well and periinterventional mortality was zero. Stent re-dilatation was always feasible and performed in 36/40 (89%) after 1 (0.8–1.5) year. Reasons for cardiac catheter reinterventions were stent outgrowth in 26 (72%), neointima formation in 8 (22%), or stent fracture in 2 (6%).

### Factors Associated with PA-Stent Implantation

Comparison between group 1 and group 2 showed, that patients with a single RV (*p* = 0.001), or a hypoplastic left heart syndrome (HLHS)-complex (*p* < 0.001), or an earlier BDCPC (*p* = 0.003), or a larger diameter of the (neo-)ascending aorta (normalized for BSA) prior to BDCPC [34.1 (31.1–43.7) versus 31.2 (25.3–35.4) mm/m^2^_;_ *p* < 0.0001] had a higher incidence of PA-stent implantation. Patients with no intervention at stage I (*p* = 0.05) or central pulmonary banding (*p* = 0.04) at stage I had a lower incidence of PA-stent implantation. All other patients’ characteristics and parameters are shown in Table 1, including the invasive hemodynamics pre-BDCPC, and these were not significantly different between the two groups.

A logistic regression was performed to ascertain the effects of the PA-dimension pre-BDCPC, diameter of the (neo-)ascending aorta pre-BDCPC, and ventricular anatomy on the likelihood of needing a PA-stent implantation after BDCPC. The logistic regression model was statistically significant, χ^2^(4) = 26.398, *p* < 0.0001. The model explained 35.0% (Nagelkerke *R*^*2*^) of the variance necessity for PA-stent implantation. Single RV patients were more likely to need a PA-stent than single left ventricle patients (OR 0.41; *p* = 0.05). Since majority of the single RV patients are HLHS, the increased incidence of LPA stent in these patients can be attributed to a natural “overcorrection” of the neo-aorta often undertaken during surgery. Smaller diameter of LPA (mm/m^2^) and larger diameter of the (neo-)ascending aorta pre-BDCPC were associated with an increased likelihood of PA-stent implantation after BDCPC (resp. OR 0.89, *p* = 0.03; OR 1.05, *p* = 0.001). Age at BDPCP did not add significantly to the model (OR 1.0, *p* = 0.61).

Based on the results of this logistic regression, we built a prediction model to estimate the predicted probability of LPA-stent implantation after BDCPC.

The equation of the model is (as described in the methods):

$${\text{predicted}}\,{\text{logit}}\, = \, - 3.010911\, + \,\left( { - 0.130738*\,\left( {{\text{LPA}}\,{\text{in}}\,{\text{mm/m}}^{2} } \right)} \right)\, + \,(0.053830*\,\left( {{\text{ascending}}\,{\text{aorta}}\,{\text{in}}\,{\text{mm}}} \right)$$

$${\text{predicted}}\,{\text{probability}}\, = \,(e^{ \wedge } {\text{Predicted}}\,{\text{logit}}){/}(1\, + \,e^{ \wedge } {\text{Predicted}}\,{\text{logit}})$$

**Fig. 3:**
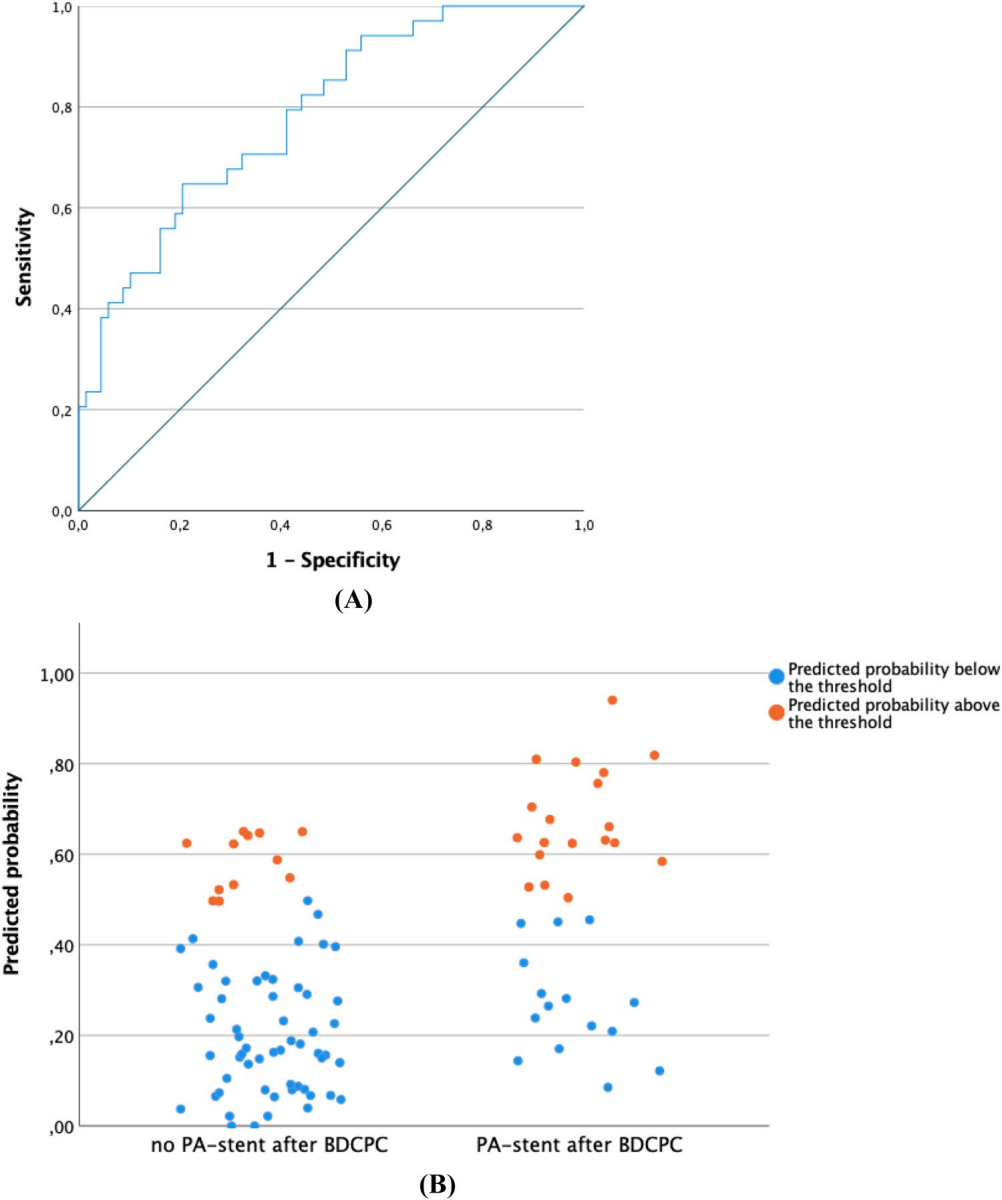
Specificity and sensibility of the predicted probability according to our prediction model on a ROC-curve (AUC = 0.78) (**A**). The threshold with the highest sensitivity and specificity was 0.45. Jittered scatter plot showing how this threshold correctly classified 65% of the patients with a PA-stent (true positives) and incorrectly included 12% of the patients without a PA-stent (false positive) (**B**)

We tested the specificity and sensibility of the predicted probability on a ROC-curve (AUC = 0.78) and according to our ROC-curve the threshold with the highest sensitivity and specificity was 0.45 (Fig. 3). This threshold (0.45) correctly classified 65% of the patients with a PA-stent (true positives) and incorrectly included 12% of the patients without a PA-stent (false positive).

### Pulmonary Artery Dimensions and Growth (Table 2* and *Fig. 4*)*

**Table 2:** Dimensions and growth of the pulmonary arteries of the complete patients’ cohort as well as comparison of group 1 (with PA-stent) and group 2 (without PA-stent) at the cardiac catheterization prior to BDCPC and TCPC

Patients			All patients		With PA-stent		Without PA-stent		
			n	%	n	%	n	%	p
			median	range	median	range	median	range	p
Cath prior to BDCPC									
Age		weeks	15	11.0; 18.9	11.2	9.2; 16.4	15.4	12.4; 19.8	**0.007**
BSA		m^2^	0.29	0.27; 0.32	0.27	0.24; 0.29	0.3	0.28; 0.32	**0.001**
(Neo)- ascending aorta		mm/m^2^	28.7	23.9; 33.8	34.15	31.1; 43.7	31.2	25.3; 35.4	** < 0.001**
Pulmonary arteries		LPA (mm)	6	4.9; 7.4	4.9	4.0; 6.1	6.3	5.25; 7.75	** < 0.001**
		RPA (mm)	5.9	4.9; 7.0	5.1	4.1; 6	6.2	5.1; 7.5	** < 0.001**
		LLL (mm)	5.1	4.4; 6.0	4.5	3.8; 5.6	5.4	4.5; 6.15	** < 0.001**
		RLL (mm)	5.3	4.2; 6.2	4.4	3.6; 5.6	5.6	4.6; 6.4	** < 0.001**
PA indexes		LPA (mm/m^2^)	20.9	17.0; 23.6	17.6	14.6; 22.5	21.3	18.2; 24.6	**0.001**
		RPA (mm/m^2^)	20	17.3; 24.1	18.5	15.9; 22.0	20.6	17.6; 24.6	**0.01**
		ratio RPA:LPA	0.98	0.88; 1.13	1	0.88; 1.17	0.97	8.87: 1.13	0.42
		ratio RLL:LLL	1	0.88; 1.1	1	0.9; 1.1	1	0.9; 1.1	0.12
		McGoon	1.75	1.49; 2.14	1.56	1.31; 1.85	1.86	1.57; 2.27	** < 0.001**
		Nakata	185	136.7; 273.7	147	110; 204	213	158; 289	** < 0.001**
		LL-index	148	148 113; 178	134	103; 178	157	122; 196	0.08
Cath prior to TCPC									
Biometrics		Age (m)	26	24; 30	29	25; 34	26	24; 29.7	0.08
		BSA (m^2^)	0.54	0.52; 0.57	0.54	0.52; 0.56	0.55	0.51; 0.57	0.799
Time from BDCPC		years	2	1.78; 2.35	1.98	1.77; 2.34	1.99	1.81; 2.4	0.1
Pulmonary arteries		LPA (mm)	6.7	0; 7.6	7.2	5.9; 8.5	8.5	7.1; 9.6	**0.004**
		RPA (mm)	7.2	6.4; 7.8	8.4	8.0; 8.8	9.3	8.2; 10.6	**0.008**
		LLL (mm)	6.7	6; 7.6	6.4	5.7; 7.1	4.7	6.1; 7.6	**0.04**
		RLL (mm)	7.2	6.4; 2.8	6.7	5.8; 7.3	5.6	6.6; 7.9	**0.005**
PA indexes		LPA (mm/m^2^)	15	12.7; 17.2	13.2	11.1; 15.6	15.8	13.0; 17.3	**0.004**
		RPA (mm/m^2^)	16.6	15; 19.0	15.7	14.2; 16.8	17.4	15.4; 19.4	**0.01**
		ratio RPA:LPA	1.13	1.0; 1.27	1.18	1; 1.43	1.11	1; 1.25	0.26
		ratio RLL:LLL	1	0.94; 1.2	1.02	0.95; 1.2	1	0.95; 1.2	0.65
		McGoon	1.9	1.55; 2.15	1.57	1.43; 1.81	2	1.7; 2.26	** < 0.001**
		Nakata	221	168; 273	179	143; 230	237	177.2; 291.5	**0.009**
		LL-index	139	120.75; 165.50	121	96; 149	141	125; 178	**0.003**
Relative growth of PA-arteries (BDCPC to TCPC)									
LPA (mm/m^2^)		%	− 28	(− 38, − 17)	− 29.6	(− 26.1,− 2.9)	− 28.8	(− 15,− 6)	0.5
RPA (mm/m^2^)	%		− 15	(− 30, − 5.9)	− 16.5	(− 26.1, − 2.9)	− 15	(− 31,− 6)	0.7
Nakata	%		12.4	(− 10, 42)	26	(− 14.2, 40.1)	7.5	(− 7,1 8.2)	0.07
McGoon	%		6.2	(− 8, 25)	3.49	(− 7.1, 18.2)	6.65	(− 9.4, 27.4)	0.8
LL-Index	%		− 8	(− 25, 25)	− 10.4	(− 28.3, 6.9)	− 7.7	(− 24, 26)	0.6
Ratio RPA:LPA	%		14.9	(− 2, 35)	14.2	(− 11.3, 6.9)	16.1	(− 5, 34.3)	0.5

Statistically significant results are given in bold

*BDCPC* bidirectional cavopulmonary connection, *BSA* body-surface-area, *RPA* right pulmonary artery, *LPA* left pulmonary artery, *LLL* left lower lobe, *RLL* right lower lobe, *LL-index* lower lobe index, *PA* pulmonary arteries, *TCPC* total cavopulmonary connection

**Fig. 4:**
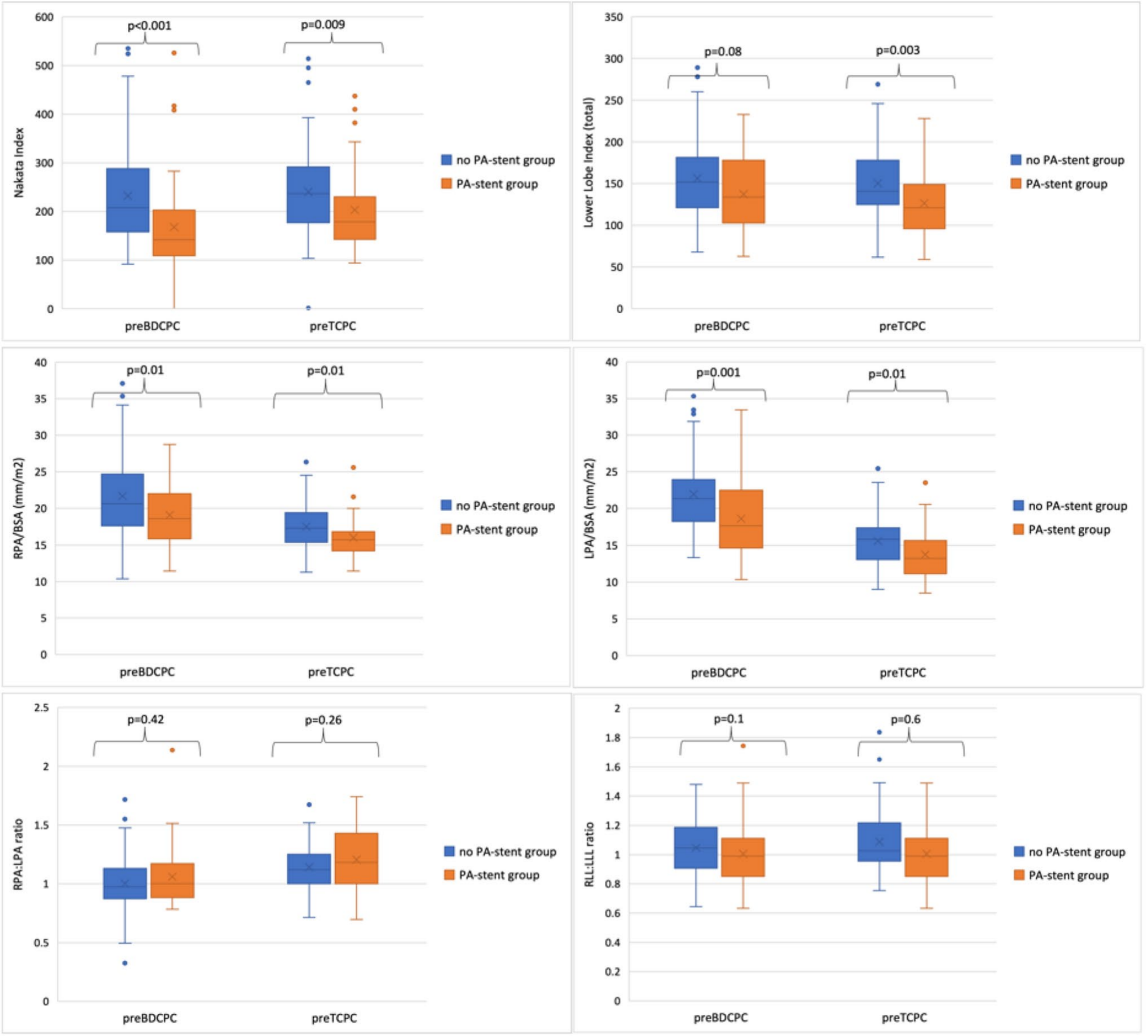
Collective pulmonary artery diameters pre-BDCPC were lower in the PA-stent group: Nakata (*p* < 0.001), RPA mm/m^2^ (*p* = 0.01), and LPA mm/m^2^ (*p* = 0.001). Lower Lobe Index was lower, but not significantly (*p* = 0.08). While absolute pulmonary arteries diameters increased in both groups, indexed pulmonary artery diameters decreased equally in both groups, but remained lower pre-TCPC in the PA-stent group: Nakata (*p* = 0.009), RPA mm/m^2^ (*p* = 0.01), and LPA mm/m^2^ (*p* = 0.01), Lower Lobe Index (*p* = 0.003). LPA and RPA grew symmetrically in both groups

At cardiac catheterization pre-BDCP the PA-stent group was younger (*p* = 0.007). Overall, pulmonary artery diameters pre-BDCPC were lower in the PA-stent group, specifically Nakata (*p* < 0.001), RPA mm/m^2^ (*p* = 0.01), and LPA mm/m^2^ (*p* = 0.001). Lower Lobe Index was tendentially lower, however not reaching statistical significance (*p* = 0.08). From the assessment pre-BDCPC and pre-TCPC absolute pulmonary arteries diameters increased in both groups but indexed pulmonary artery diameters decreased in both groups. The individual relative growth (%) was not different within the groups for all parameters. At cardiac catheterization pre-TCPC age was similar between the groups. Nakata (*p* = 0.009), RPA mm/m^2^ (*p* = 0.01), and LPA mm/m^2^ (*p* = 0.01), and Lower Lobe Index (*p* = 0.003) remained lower pre-TCPC in the PA-stent group. RPA:LPA ratio and LLL:RLL ratio remained symmetric at both assessments between and within the groups.

## Discussion

This investigation is one of the first studies analyzing pulmonary artery growth pattern between the time of BDCPC and TCPC focusing on the potential beneficial impact of central pulmonary artery stenting [7]. Our data show that PA stenting becomes frequently necessary, as shown by others [5–10], and may ensure symmetric PA growth comparable with the non-stented PA side branch.

Factors associated with need for central PA stenting may be predicted before BDCPC, especially for the LPA, which is predominantly affected. This includes smaller indexed diameter of LPA pre-BDCPC, larger diameter of the (neo-) ascending aorta pre-BDCPC, and cardiac anatomy consistent with HLHS-complex. These predictors align with the intraprocedural findings, according to which a compression from the (neo-) ascending aorta presumably is the main cause of LPA stenosis, whereas two of three patients that underwent RPA-stenting had a right aortic arch.

Pulsatile compression from a larger (neo-) ascending aorta is an important anatomic feature for potential postoperative LPA compression [7, 8, 11, 12]. Especially in HLHS-complex patients the position of the Damus-Kaye-Stansel anastomosis moves the ascending aorta more to the left in comparison to patients with a native aortic arch, while the reconstruction of the inner curvature of the aortic arch with xenopericardium may lead to foreshortening over time and thus decreases the space under the arch through which LPA and left bronchus pass. These could contribute to LPA—and bronchus—compression [1].

The surgical approach with a comprehensive stage I and II had been associated with higher incidence of PA-interventions [8, 9], but this was not a significant risk in our cohort. Further on, earlier BDCPC seems to affect only marginally PA growth. All other assessed anatomical and surgical factors are not associated with an increased risk for PA-stent implantation after BDCPC.

SV patients, depending on their cardiac anatomy, might have small pulmonary arteries directly after birth [2–4]. During the interval between stage I and II they can have a “catch-up” growth due to pulmonary overflow and pulsatile pulmonary blood flow (Sano shunt), while after stage II they normally have an inadequate development of the pulmonary vasculature, that could lead to increased pulmonary vascular resistance [2–4]. BDCPC creation from a shunted single ventricle leads to volume unloading, which may lead to under-filling of the branch PAs. These considerations are in line with the relative decrease of the size of PAs between BDCPC and TCPC found in our analysis and previous assessments in literature [23–25].

Since the growth of the PAs depends on blood flow [26], improvement in the caliber of the LPA by PA-stent implantation could potentially maximize future growth [7]. Currently, there is limited data published on distal PA growth after PA-stent implantation. One single analysis [19] on 18 SV patients showed, a similar growth of the stented and non-stented contralateral pulmonary artery branches, which is in line with our findings. In our cohort, we could also show that the growth of the PA-system is not only symmetric with the contralateral side, but also similar to the group without a PA-stent. As expected, a PA-stent implantation does not help to fully “catch-up” in growth, but ensures a PA growth comparable to non-PA stented patients [2–4]. However, other studies have shown direct positive implications of a LPA-stent implantation. In fact, an increase in cardiac output associated with LPA-stenting in SV patients has been demonstrated [27], while MRI-based studies have shown, that zones of narrowing within the non-pulsatile pulmonary artery circuit relate to relevant energy losses in proportion to the degree of narrowing [1].

It is well reported in literature, that early postoperative cardiac catheterization after surgery for congenital heart disease is feasible and safe, even when acting on freshly formed sutures or hemodynamic instable patients [5, 12, 14–17]. Stent implantation to treat PA stenosis in SV patients is effective and can be realized safely [7, 10]. In other cohorts [15, 27, 28], angioplasty was performed safely on fresh suture lines without reported vascular tear or suture disruption. In our cohort, one patient probably experienced a suture disruption, but this was related to the balloon-angioplasty prior to LPA-stent implantation. This is concordant with reports from Zahn et al. [29], which concluded that continuous Prolene suture lines can be expanded safely without disruption when using balloon to stenosis ratio ≤ 2.5/1 [29]. In our cohort, we used a stent-to-stenosis ratio of 2.4 (IQR 1.9–3.7). Nevertheless, a not negligible periprocedural risk profile of PA stenting early after BDCPC remains and an immediate surgical backup dealing with severe PA bleeding complications is necessary [17].

The selection of a particular stent is mainly depended on the size of the reference vessels [29, 30], the characteristics of the stenosis [13], and the size of the patient with regards to the sheath size needed for implantation. The initial diameter of the stent was chosen conservatively without overdilating the stenosed area. Since the rate of vessel complications associated with PA-stenting is low, bare metal (instead of covered) stents were used.

Since stents implantation was performed in growing vessels, repetitive stent re-dilatations were performed to accommodate the vessel diameter for somatic growth. Stent re-dilatation was always feasible [31].

When performing stent implantation stents that can be dilatated to reach the adult diameter of the pulmonary arteries (ca. 15 mm) should be preferred [32]. Until now, all available stents have limited final expansion diameters. Breakable stents, such as the Bentley BeGrow™ stents, could be a viable option in some cases since they can be implanted in small vessels (4-6 mm) with a 4 French sheath but have the potential for further re-dilations and controlled stent strut breakage to fit with the growth of the contralateral pulmonary branch [22]. Bioabsorbable stents could also potentially allow further growth of the artery and remodeling of the vessel wall [33].

A model for prediction of the necessity for an LPA-stent implantation after BDCPC would be an appealing tool for the clinician and could potentially lead to a change in the clinical management of these patients. In fact, our prediction model might estimate the probability of the necessity for PA-stent implantation and could be used to (a) perform a close follow-up or (b) plan an exit-angiography directly after BDCPC in patients at risk for pulmonary artery complications or, in high-risk patients, (c) perform a hybrid procedure for LPA stenting during BDCPC surgery.

In summary, a stent implantation (most often for external LPA compression), when clinically indicated, ensures symmetrical pulmonary flow and growth of the hilar and intrapulmonary vessels. However, side branch PA stents require multiple catheter interventions to match the child’s growth.

### Limitation

Our study is a retrospective study with its inherent limitations. A group comparison with patients that had a stenosis in the PA-system but did not receive a PA-stent is impossible from an ethical perspective. The growth of the pulmonary arteries is influenced by many other factors, such as the blood flow distribution to the left and right PA, that we did not assess. The direct clinical impact of the PA-implantation is hard to define and therefore not assessed in this analysis. The aim of our study focused on the time frame between BDCPC and TCPC rather than long-term outcome. Further surgical factors such as placement of the pulmonary artery confluence to the left or right of the midline [1, 34] could not been evaluated. The architecture of the aortic arch other than the dimensions of the ascending aorta has not been evaluated (34). Lastly, our prediction model is based on the results of our institution, and it might not fit for another institution with a different surgical or interventional strategy.

## Conclusion

PA-stent implantation early after BDCPC is feasible and safe. Single RV, larger (neo-) ascending aorta, and smaller LPA prior to BDCPC are independent risk factors for PA-stent implantation early post-BDCPC. Our results suggest a pre-BDCPC risk-stratification for PA-stent implantation is theoretically feasible and at least an exit-angiography should be discussed in risk cases. PA diameters after PA-stent implantation and stent dilatation show symmetric but limited growth together with the contralateral side, similar to the no-PA-stent group. Therefore, PA-stents do not negatively influence peripheral PAs growth and should not be withheld, if clinically indicated.

## Data Availability

The authors confirm that the data supporting the findings of this study are available within the article. Supplementary data that support the findings of this study are available from the corresponding author, AC, upon reasonable request.
